# Contribution of Macrophages and T Cells in Skeletal Metastasis

**DOI:** 10.3390/cancers12041014

**Published:** 2020-04-20

**Authors:** Veronica Mendoza-Reinoso, Laurie K. McCauley, Pierrick G.J. Fournier

**Affiliations:** 1 Department of Periodontics and Oral Medicine, University of Michigan School of Dentistry, Ann Arbor, MI 48109, USA; verom@umich.edu (V.M.-R.); mccauley@umich.edu (L.K.M.); 2 Department of Pathology, University of Michigan Medical School, Ann Arbor, MI 48109, USA; 3 Biomedical Innovation Department, Centro de Investigación Científica y de Educación Superior de Ensenada, Ensenada, BC 22860, Mexico

**Keywords:** bone metastasis, TAMs, MAMs, macrophage polarization, efferocytosis, T cells, immunosuppression, immunotherapy

## Abstract

Bone is a common site for metastases with a local microenvironment that is highly conducive for tumor establishment and growth. The bone marrow is replete with myeloid and lymphoid linage cells that provide a fertile niche for metastatic cancer cells promoting their survival and growth. Here, we discuss the role of macrophages and T cells in pro- and anti-tumoral mechanisms, their interaction to support cancer cell growth, and their contribution to the development of skeletal metastases. Importantly, immunotherapeutic strategies targeting macrophages and T cells in cancer are also discussed in this review as they represent a great promise for patients suffering from incurable bone metastases.

## 1. Introduction

Skeletal metastasis affects the quality of life in cancer patients and it is commonly associated with significant morbidity. In the bone marrow, various types of immune cells such as macrophages, T cells, natural killer cells, dendritic cells, myeloid-derived suppressor cells, and neutrophils have been identified as contributors to the development of skeletal metastases [1,2,3,4,5,6]. In most cancers, tumor associated macrophages (TAMs) represent up to 50% of the tumor mass and their high density is directly related to poor prognosis [7,8]. TAMs have been associated with the development of the metastatic cascade in breast and prostate cancers [9,10]. Macrophages are commonly described to be polarized towards M1 (classically activated, pro-inflammatory) or M2 (alternative activated, anti-inflammatory) phenotypes and they acquire different functional programs depending on the signals present in the tissue microenvironment [11]. The M1 phenotype is induced IFN-γ, LPS, and TNF-α. However, M2 macrophages have three forms: M2a or “alternative” induced by IL-4 and IL-13, M2b induced by immune complexes and agonists of Toll-like receptors or IL-1R, and M2c induced by IL-10 and glucocorticoid hormones. Each of these M2 macrophages forms releases a different set of chemokines that recruit a distinctive group of immune cells that promote different immune responses [12,13]. TAM polarization towards the immunosuppressive and pro-tumorigenic M2 phenotype has been demonstrated in various studies [11,14,15]. Interestingly, around 60% of total macrophages in the normal bone microenvironment are polarized towards the M2 phenotype, suggesting that metastatic cancer cells encounter a pro-tumoral environment in the bone [16]. Moreover, a newly identified population of macrophages, named metastatic-associated macrophages (MAMs), has a specific role in metastasis development [17]. Conversely, the presence of tumor infiltrating lymphocytes (TILs), particularly CD8^+^ T cells, is a marker for a favorable prognosis in cancer patients [18]. However, in chronic inflammatory processes such as cancers, T cells become dysfunctional due to persistent antigen exposure [19,20] and fail to remove cancer cells effectively [21]. Constant antigen stimulation in cancer promotes the expression of immune checkpoints such as the programmed cell death protein 1 (PD-1), which contributes with the inhibition of the immune response [22]. In the bone marrow, about 1.5% of T cells are CD4^+^ and 2.0–2.5% are CD8^+^ [23,24]. Tregs are CD4^+^ T cells known as immune suppressors that are activated in cancer tumors, which reduce the immune response against cancer cells [1].

**Figure 1:**
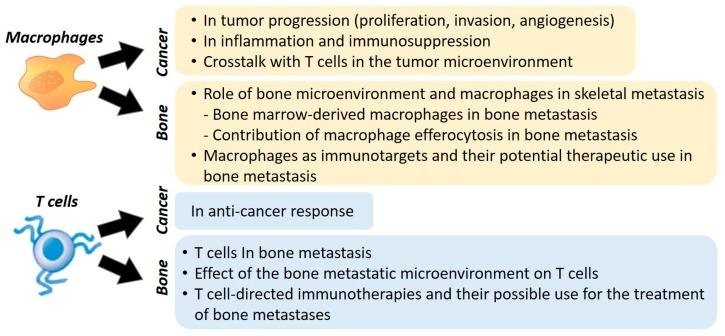
Topics covered in this review.

In this review, we discuss the function of TAMs, MAMs, and T cells in the development of metastases. Most importantly, we report the current therapeutic strategies targeting macrophages and T cells in tumor progression and how these strategies may be used for the treatment of bone metastases (Figure 1).

## 2. Macrophages

**Figure 2:**
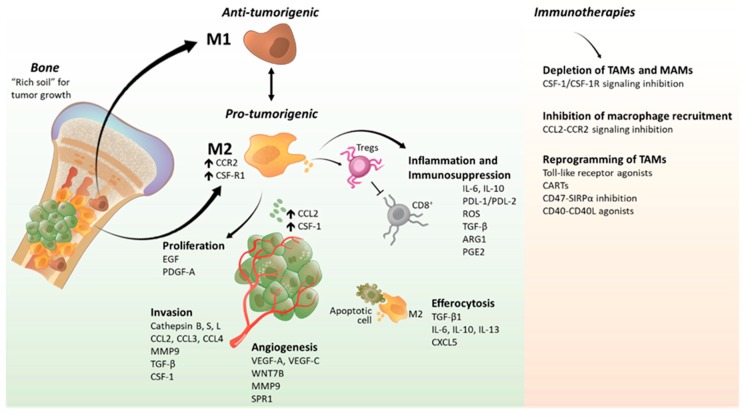
The role of macrophages in tumor development and bone metastases. The bone microenvironment is a “rich soil” for tumor growth. Tumor associated macrophage polarization towards the M2 phenotype promotes the development of the metastatic cascade that contributes to the development of skeletal metastases once the bone is colonized by metastatic cancer cells. Various immunotherapeutic strategies are currently under clinical trials in order to find effective treatments.

Macrophages are specialized innate immune cells of the myeloid lineage that are responsible for engulfing and digesting pathogens, apoptotic cells, and cell debris. Due to their diversity and plasticity, macrophages have multiple key roles in the regulation of immune responses, inflammation, and tissue homeostasis. Macrophages are widely distributed in all tissues and mainly originate from bone marrow-derived monocytes or tissue-resident macrophages derived from yolk sac progenitors [25,26]. During primary and metastatic tumor development, acute inflammatory processes recruit macrophages to the tumor microenvironment; these macrophages are known as tumor-associated macrophages (TAMs) and associate with poor prognoses in most solid cancers [11,14,27]. Inflammatory cytokines produced in the tumor microenvironment can polarize M1 macrophages (tumor suppressors) to the M2 subtype (immune suppressors). The majority of macrophages present in the tumor microenvironment are recruited from bone marrow-derived monocytes through the CC chemokine 2 (CCL2/CCR2) [28,29,30,31] and colony stimulating factor 1 (CSF-1/CSF-1R) [32] signaling pathways. However, studies have identified a mixture of yolk sac-derived and bone marrow-derived macrophages in the tumor microenvironment suggesting that, depending on their origin, TAMs have different roles during tumor progression [33,34,35]. TAMs secrete inflammatory cytokines, growth factors and proteolytic enzymes that promote cell proliferation, invasion, and angiogenesis [7,36,37]. TAMs also contribute to cancer development and metastasis by increasing inflammation and suppressing the T cell immune response through the production of cytokines and negative checkpoint regulators (Figure 2) [14,38].

In the bone, osteal macrophages play a pivotal role in the regulation of bone formation and skeletal homeostasis [39,40]. Different types of tumors are capable of spreading to the bone; however, prostate, breast, and lung cancer are responsible for the majority of skeletal metastases [41]. Once tumor cells localize in the bone microenvironment, they land in a richly myeloid cell milieu that contributes to metastatic tumor progression [4,42]. Few studies have suggested that TAMs have a positive role in the regulation of bone metastases based on their ability to promote the entrance of cancer cells to organs through capillaries [43]. In addition, it has been shown that inhibition of macrophage recruiting factors and TAM reprogramming from M2 to M1 reduce bone tumor growth [42,43]. However, the exact TAM molecular mechanisms in the promotion of skeletal metastasis remain to be elucidated. The phagocytic clearance of apoptotic cells is known as efferocytosis. Recently, macrophage-dependent efferocytosis of apoptotic cancer cells was identified in a mechanistic pathway promoting tumor progression and metastasis [44].

### 2.1. Role of Macrophages in Cancer

#### 2.1.1. Tumor-Associated Macrophages in Tumor Progression and Metastasis

##### TAMs in Cancer Cell Proliferation

Sustained tumor cell proliferation is key during cancer development and progression. Tumor cell proliferation is accomplished by dysregulation of the cell cycle and the constitutive activation of various signal transduction pathways that stimulate cell growth. In most cancers, proliferative processes are mainly regulated by signaling proteins released by cells present in the tumor microenvironment. TAMs are M2-like polarized macrophages that secrete cytokines, chemokines, and growth factors in the tumor microenvironment to promote tumor cell growth and metastasis [45,46,47]. One of the growth factors secreted by TAMs is the epidermal growth factor (EGF), which stimulates the expression of its receptor (EGFR) in breast cancer cells to continuously activate the signal transducer and activator of transcription 3 (STAT3)/SRY-Box Transcription Factor 2 (Sox-2) signaling pathway enhancing cancer cell survival and proliferation in mice [48,49,50]. This was confirmed by another study where the authors demonstrated that CD206^+^ (M2-like) TAMs secrete high levels of EGF in oral squamous cell carcinomas (OSCC), and that cell proliferation was increased in OSCC cells treated with conditioned media from CD206^+^ TAMs [51]. Further studies demonstrated that EGF produced by M2-like TAMs suppresses long non-coding RNA inhibiting metastasis (LIMT) expression to promote proliferation, migration, and invasion in ovarian cancer through the activation of EGFR-extracellular-signal-regulated kinase (ERK) signaling [52]. Previous studies found platelet-derived growth factor (PDGF) enhanced cancer cell proliferation [53]. Interestingly, a study evaluated non-small cell lung cancer tumors and found TAMs express PDGF-A and -B chain genes, whereas mesenchymal and endothelial cells expressed PDGF receptor. These findings established a correlation between the strong expression of PDGF-A and -B chains genes by TAMs and high replication rates of mesenchymal and endothelial cells [54]. Hence, M2 polarization of TAMs in the tumor microenvironment presents unique regulation of mitogenic processes to promote cancer cell proliferation.

##### TAMs in Cancer Cell Invasion

Metastasis is a life-threatening event for cancer patients. The invasive potential of cancer cells relies on cell–cell and cell–matrix adhesion changes as well as the intercellular paracrine signals in the tumor microenvironment. Cathepsins modify the extracellular matrix to promote basal membrane dysfunction during tumor cell invasion and metastasis. In vitro and in vivo experiments have demonstrated that cathepsins B and S improved the invasiveness of pancreatic cancer cells [55]. Further studies revealed that STAT3 and STAT6 promote cathepsin secretion by macrophages [56]. Recently, a study showed that inhibition of cathepsin L secretion by TAMs and breast cancer cells impairs invasion and M2-like TAM infiltration in the tumor microenvironment [57]. The macrophage inflammatory proteins 1α (MIP-1α or CCL3) and -β (MIP-1β or CCL4) are chemokines of the CC superfamily derived from immune cells, including macrophages. The expression of TAM-derived MIP-1β or CCL4 chemokine relates to poor survival in breast cancer patients, potentiating breast cancer cell invasion and metastasis through increased myosin IIIA (MYO3A) gene expression [58]. A different MIP subunit, MIP-3α, was expressed by pancreatic cancer cells and TAMs to regulate tumor cell invasion [59]. Later studies found that MIP-3α induced matrix metallopeptidase 9 (MMP9) expression via its receptor chemokine receptor 6 (CCR6), which increased pancreatic cancer cell invasion [60]. In correlation with the last study, high levels of TAM-derived MMP9 and other factors such as vascular endothelial growth factor (VEGF), chitinase 3 like 1 (CHI3L1) and lipocalin 2 (LCN2), promoted breast cancer metastasis in vivo [61]. Depending on the cellular context, transforming growth factor β (TGF-β) can control cancer cell transcriptional activities that promote the epithelial–mesenchymal transition to facilitate tumor metastasis and invasion [62,63,64]. TGF-β produced by TAMs increased SRY-Box Transcription Factor 9 (SOX9) expression via the C-jun/SMAD3 pathway, which promoted cancer cell invasion in lung cancer metastasis [65]. The monocyte chemoattractant protein 1 (MCP-1 or CCL2) is a potent chemoattractant for monocytes and other immune cells to promote their recruitment to sites of tissue injury and inflammation. CCL2 activates CCR2 and CCR4 receptors [66,67]. CCL2–CCR2 signaling was found to regulate cellular adhesion and motility in macrophages [68]. In prostate cancer, invasion of tumor cells was promoted by TAMs via CCL2-CCR2 signaling [69]. Further studies demonstrated that CCL2 induces CCL22 and CCR4 secretion in tumor cells stimulating migration and invasion of prostate cancer cells [70]. Colony-stimulating factor 1 (CSF-1) is a key regulator of TAM recruitment, differentiation, and survival [71,72]. TAMs increase tumor cell migration and invasion through a paracrine loop which consists of macrophage-derived EGF and tumor-induced CSF-1 [73]. These two factors work synergistically to induce extracellular matrix remodeling to form invadopodia and podosome formation during cancer cell invasion [74].

##### TAMs in Angiogenesis

Metastatic tumors require oxygen and nutrients to maintain their progression. Formation of blood vessels is fundamental to provide oxygen and nutrients during cancer cell proliferation. Vascularization is associated with tumor growth and metastasis, and plays a key role during cancer progression. Macrophages are instrumental in supporting a blood supply for tumor progression. Macrophages are recruited and reprogrammed by chemokines, cytokines, and growth factors secreted by tumor cells to mediate angiogenic properties [75,76]. Various cancer animal models have demonstrated that M2-like TAMs promote tumor angiogenesis in melanoma, breast, and prostate cancer [77,78,79]. These data correlate with various human cancers where higher TAM infiltration resulted in increased angiogenesis [80,81,82]. Vascular epithelial growth factor A (VEGF-A) is a pro-angiogenic cytokine released by M2-like TAMs, and high VEGF-A levels correlate with TAM density in various cancers [80,83]. Moreover, TAM-derived VEGF contributes to tumor neovascularization [84,85]. In breast cancer, TAM-secreted wingless-type MMTV integration site family, member 7B (WNT7b) increased VEGF-A expression in vascular endothelial cells to promote angiogenesis [86]. High levels of TAM-secreted VEGF-C promotes lymphangiogenesis in Merkel cell carcinoma [87]. TAMs produce MMP-9 to stimulate angiogenesis in melanoma [88] and highly expresses angiopoietin receptor 1 (TIE2) to promote tumor angiogenesis in various mouse models [89]. Recently, a group found that the sphingosine-1-phosphate receptor (S1PR1) on TAMs stimulates lymphangiogenesis and pulmonary metastasis via NLR Family Pyrin Domain Containing 3 (NLRP3)/Interleukin 1 β (IL-1β) in a breast cancer model [90].

#### 2.1.2. Role of Metastasis-Associated Macrophages in Metastatic Progression

Metastatic cancer cells need to evade the immune system, survive in the blood circulation, and reach distant sites to grow in different types of environments. The presence of TAMs in the primary tumor contributes to the progression of metastatic processes [91]. In addition, recent studies have identified a different population of macrophages in metastatic tissues, termed metastasis-associated macrophages (MAMs) [29,92].

The difference between TAMs and MAMs resides in their origin: TAMs originate from resident macrophages in the primary tumor, whereas MAMs arise from inflammatory macrophages present in metastatic sites [17,29]. Interestingly, MAMs, resident macrophages, and TAMs have been reported to be genotypically different [93]. MAMs are described to be essential to promote extravasation, secretion of growth factors, and suppression of T cell anti-tumoral responses [17,29]. Interestingly, MAM precursors represent around 80% of metastatic lung tumor mass, whereas regular monocytes represent around 5% [93]. Thus far, MAMs have been found in visceral metastatic tissues such as lung, liver, kidney, spleen, and brain [42].

The recruitment of bone marrow-derived macrophages (BMDMs) promotes a pro-inflammatory environment in the metastatic niche to allow metastatic cancer cell establishment, support of tumor growth, and further recruitment of BMDMs, which sustain the formation of metastatic tumors [93]. In addition, it has been shown that MAMs derived from BMDMs support circulating cancer cell adhesion and migration to the metastatic niche by interactions between MAMs and cancer-associated fibroblasts (CAFs) or endothelial cells [91]. The bone marrow is rich in resident macrophages; hence, the bone tissue is a favorable microenvironment for the conditioning and formation of the metastatic niche where BMDMs may contribute enormously to the enrichment of the pro-tumorigenic MAM population in bone. Further studies are needed to identify the distinctive MAM population in skeletal metastases.

MAMs express CD11b, vascular endothelial growth factor receptor 1 (VGFR1), C-X-C Motif Chemokine Receptor 3 (CXCR3), and CCR2 and interact with metastatic cells through vascular cell adhesion protein 1 (VCAM1) to promote epithelial to mesenchymal transition by producing TGF-β to support lung metastasis in mouse models [17,94,95,96,97]. MAMs also display specific polarization depending on the metastatic site, for example, in intracranial breast cancer metastases, where in the brain parenchyma, MAMs were polarized towards the M2 phenotype [98].

Inhibition of MAM recruitment is a therapeutic approach for metastases treatment. For example, a CCL2 antibody has been used to neutralize CCL2 secreted by cancer and stromal cells to inhibit the recruitment of inflammatory monocytes and MAM accumulation in breast-tumor metastases [29]. Moreover, discontinuation of CCL2 inhibitory treatment resulted in increased metastases in various mouse models of metastatic breast cancer [99]. Interestingly, a subpopulation of VGFR1+ MAMs were found to be highly angiogenic in a metastatic liver mouse model. This was in line with a high percentage of VEGFR1^+^ cells found in liver metastasis patients, which correlated with worse patient outcome [100]. Conversely, a study found that calveolin-1 (Cav1) in MAMs restrains VEGF-A/VEGFR1 signaling; therefore, Cav1 depletion in MAMs promoted an increased metastatic growth and angiogenesis in lung metastatic tissue [101].

Altogether, these studies show that therapeutic strategies designed to target MAMs in metastatic lesions are encouraging; however, further studies are required to identify the MAM-dependent molecular mechanisms to regulate metastases.

#### 2.1.3. Tumor-Associated Macrophages in Inflammation and Immunosuppression

Chronic inflammation in the tissue microenvironment increases the risk of cancer initiation and progression [102,103,104]. M1 macrophage polarization is stimulated by cytokines such as lipopolysaccharide (LPS), Interferon-gamma (IFN-γ), and tumor necrosis factor-alpha (TNF-α), and promotes inflammation and antitumor activity, whereas M2-like macrophages are stimulated by interleukin 4 (IL-4) and IL-13, and induce immunosuppression and anti-inflammatory responses [28]. About 50% of the tumor mass are TAMs [7]. TAMs secrete inflammatory cytokines and factors such as interleukin 6 (IL-6), interleukin 10 (IL-10), and TGF-β in the tumor microenvironment, which facilitate the inflammatory microenvironment to promote cancer progression and metastasis. IL-6 is a pro-inflammatory cytokine that has a pivotal role during chronic inflammation and cancer development [105,106]. High levels of IL-6 are related to tumor aggressiveness and poor response to therapies [107,108]. In the tumor microenvironment, IL-6 is mainly secreted by TAMs and is associated with tumor progression and invasion [109,110]. IL-6 activates the STAT3 signaling pathway to promote the expression of CD44^+^ in hepatocellular carcinoma cells and it increases sphere formation when cancer cells are co-cultured with macrophages [111]. Similarly, TAM-secreted IL-6 activates the STAT3 pathway in breast cancer to upregulate TGF-β1 and hypoxia inducible factor 1-alpha (HIF-1α) gene expression during chemotherapy-induced apoptosis [112]. Using glioma stem cells, IL-6 secretion by macrophages was found to promote proliferation via MYD88 innate immune signal transduction adaptor (MyD88)-toll-like receptor 4 [113]. It is well known that IL-10 is a potent anti-inflammatory cytokine and it has an important role in controlling immune responses. On the contrary, when IL-10 is secreted by M2-like TAMs in co-culture with pancreatic cancer cells, it promotes epithelial–mesenchymal transition of cancer cells via toll like receptor 4 (TLR4)/IL-10 signaling [114]. Similarly, M2-like TAM-secreted TGF-β induces epithelial–mesenchymal transition of murine hepatocytes to induce their change to stem-like cells [115].

The immunosuppressive role of TAMs has been a significant focus. Studies suggest that M1-like macrophages are predominant in most of the early tumor stages, whereas M2-like TAMs are typically recruited in hypoxic areas of advanced tumors [116]. In the tumor microenvironment, immune checkpoint molecules such as the programmed cell death protein ligands (PD-L1/PD-L2) are highly expressed by TAMs. These ligands bind to the program cell death protein-1 (PD-1) to inhibit T-cell activation and effector functions, therefore suppressing their immune response against cancer cells [117,118,119]. Recently, a study found that macrophages treated with an anti-PD-L1 antibody increased macrophage proliferation and their pro-inflammatory response, however, treatment with PD-1 and PD-L1 neutralizing antibodies resulted in decreased tumorigenesis in a B16 melanoma animal model [120]. Elevated rates of reactive oxygen species (ROS) have been detected in various cancers, where they promote tumor development and progression. One study demonstrated that ROS regulates PD-L1 expression by macrophages and their immunosuppressive and pro-angiogenic signaling [121]. Conversely, another study characterized the immune signature of PD-L1^+^ tumors and identified that the balance of TAMs and T cells in PD-L1^+^ tumors regulates the disease outcome in cancer patients. In addition, the inflammatory signaling from TAMs produced PD-L1^+^ cancer cells that supported angiogenesis and metastasis, whereas PD-L1^+^ cancer cells generated by activated T cells are sensitive to therapy [122]. M2-like TAMs secrete strongly immunosuppressive cytokines such as IL-10, TGF-β, arginase-1 (Arg1), and prostaglandins to inhibit the activity, proliferation and antitumorigenic effects of T-cells in the tumor microenvironment. An in vitro study found increased IL-10 (immunosuppressor) and decreased IL-12 (CD8^+^ T cell proliferation and effector function enhancer) production by TAMs that promoted a pro-tumorigenic phenotype [123,124]. Later on, a study isolated TAMs derived from primary lung cancer tissues and found a strong correlation between increased *IL-10* and stage, tumor size, lymph node metastasis, and lymphovascular invasion in non-small cell lung cancer patients [125]. Moreover, it has been shown that TAMs were the primary source of IL-10 in mammary mouse tumors, which caused the inhibition of CD8^+^ T cell-dependent responses. In the same study, IL-10 receptor blockade increased IL-12 expression in intratumoral dendritic cells, which was associated with reduced tumorigenesis [126]. TAMs secrete high amounts of TGF-β, which promotes their own M2 polarization to enhance immunosuppression [127]. TGF-β stimulates interleukin 1 receptor associated kinase M (IRAK-M), a toll-like receptor signaling inhibitor, expression in TAMs to promote immune evasion in lung tumors [128]. Further studies demonstrated that TGF-β induces M2-like tryptophan hydroxylase 1 (TPH-1) macrophages via zinc finger proteins (SNAIL) upregulation depending on the SMAD2/3 and PI3K/AKT signaling pathways [129]. M2-like TAMs are characterized for having high expression levels of arginase 1 [130]. An in vivo study identified higher numbers of the immunosuppressive Arg1^+^ macrophages in tumors and showed that anti-programmed cell death-1 (anti-PD-1) treatment diminishes Arg1^+^ and increases Arg1- TAMs in the tumor microenvironment [131]. Interestingly, a study demonstrated that the COX2/mPGES1/PGE2 pathway regulates PD-L1 expression in TAMs to promote prostaglandin E2 (PGE2) metabolism and immunosuppression [132]. Consequently, these studies provide evidence that TAMs mediate chronic inflammatory processes and immunosuppressive functions to support tumor growth and pro-metastatic mechanisms.

#### 2.1.4. Crosstalk between Macrophages and T-Cells in the Tumor Microenvironment

During tumor immune surveillance, CD8^+^ cytotoxic T cells have an essential role promoting tumor cell death [133]. However, in most cancers, the tumor microenvironment is infiltrated by TAMs that, in cooperation with regulatory CD4^+^ T cells, creates an immunosuppressive microenvironment and inhibits the activated T effector cells [134]. It is well known that M2-like TAMs play a crucial role during immunosuppression [135]. Interestingly, a study showed that CD8^+^ T cell depletion from squamous cell carcinoma tumors correlates with low lymphocyte motility and poor outcome. TAMs interact with CD8^+^ T cells to trap them in the tumor stroma and TAM depletion using a CSF-1R inhibitor increased CD8^+^ T cell migration and infiltration into tumors [136]. Regulatory T cells (Tregs) are known as immunosuppressive cells in the tumor microenvironment [137]. Recently, it was demonstrated that Tregs inhibit the production of IFN-γ by CD8^+^ T cells and increase sterol regulatory element-binding protein 1 (SREBP1)-dependent lipid metabolism in TAMs to promote the immunosuppressive M2-like TAM phenotype in B16 melanoma and MC38 colon adenocarcinoma tumor models [138]. In glioblastoma, activation of the aryl hydrocarbon receptor (AHR) by dysregulation of the kynurenine pathway contributes to the malignant properties of these tumors. A study showed that AHR promotes the expression of CD39 in TAMs to drive CD8^+^ T cell dysfunction during the immune response in the tumor microenvironment [139].

Altogether, these studies confirm that therapeutic targeting of TAMs is a promising strategy for cancer treatment. Molecules that target M2-like TAMs exclusively would be prudent since M1 macrophages are essential to promote the T cell immune response.

### 2.2. Role of Bone Microenvironment and Macrophages in Skeletal Metastasis

Osteal macrophages or osteomacs are macrophages that reside in bony tissues and have a crucial role during bone formation and remodeling. About 16% of total isolated calvarial cells correspond to mature macrophages (F4/80+) [39,140]. Osteomacs or resident macrophages in bone, are distributed on bone surfaces intercalated within resting osteal tissue and immediately adjacent to mature osteoblasts where bone remodeling takes place [39]. Interestingly, over 75% of osteoblasts on the endosteal surface of cortical bone are covered by osteal macrophages [40]. During bone regeneration, osteoblasts undergo apoptosis and macrophages recruited from the bone marrow phagocytose apoptotic osteoblasts, a process known as efferocytosis, in order to maintain normal bone homeostasis [140]. When tumors metastasize to bone, they encounter robust numbers of bone marrow myeloid lineage cells and osteal macrophages. Interestingly, a recent study found that bone marrow-derived but not peritoneal macrophages have a very distinctive pro-inflammatory response upon efferocytosis of apoptotic cancer cells, which may support the development of skeletal bone metastasis [16].

#### 2.2.1. Bone Marrow-Derived Macrophages in Bone Metastasis

Breast and prostate cancer patients often develop bone metastasis [141]. The “seed and soil” hypothesis highlights that the specific organ microenvironment plays a critical role in the development of metastasis. To form bone metastases, cancer cells from the primary tumor have to go through the metastatic cascade that includes invasion of surrounding tissues, intravasation, migration, survival in the blood stream, extravasation, angiogenesis, and pre-metastatic niche formation. TAMs are key components during primary tumor progression and the development of the metastatic cascade producing or promoting the secretion of inflammatory and immunosuppressive proteins as described in this review.

Bone metastases are classified as osteolytic, osteoblastic, or mixed. Osteolytic bone metastasis is characterized by the destruction of normal bone mediated by osteoclasts [142]. Parathyroid hormone-related peptide (PTHrP) and the receptor activator of NF-kappaB ligand (RANKL) are crucial during the development of osteolytic lesions [143,144,145]. On the other hand, osteoblastic or sclerotic lesions are characterized by new bone deposition where transforming growth factor, bone morphogenic proteins (BMP), and endothelin-1 are associated with osteoblast generation [146].

#### 2.2.2. Contribution of Macrophage Efferocytosis in Bone Metastasis

Efferocytosis is the clearance of apoptotic cells by phagocytic cells such as macrophages. This process has a key role in maintaining normal tissue homeostasis. Efferocytosis is regulated by “find me” and “eat me” signals. Soluble factors such as CCL2 and CXCL1 are chemokines [147] that recruit macrophages; then, apoptotic cells expose plasma membrane markers, such as phosphatidylserine, to allow macrophages to recognize them, bind to the dying cell, and initiate the engulfment process [148,149]. During tumor progression and cytotoxic treatments, millions of cancer cells undergo cell death. Under normal circumstances, efferocytosis is a crucial process that prevents tissue inflammation; however, the clearance of dying cells’ remains by M2-like TAMs in the tumor microenvironment promotes immunosuppression through the inhibition of CD4^+^ and CD8^+^ T cells [150]. Inhibition of efferocytosis through the blockade of “eat me” signals reduces tumor progression and metastasis in various types of cancers [45,151,152,153].

A recent study demonstrated that efferocytosis of apoptotic cancer cells activates NF-κB and STAT3 transcriptional machinery to promote the production of the pro-inflammatory C-X-C motif chemokine 5 (CXCL5) in the bone microenvironment, which supported prostate cancer skeletal metastasis [44]. Similarly, another study found that tumor cell debris, after cancer therapy, promotes survival and growth of living cancer cells in multiple tumors through the secretion of pro-inflammatory cytokines such as TNF-α, IL-6, IL-8, CCL4, and CCL5 by macrophages [154]. Interestingly, a study has demonstrated that macrophage efferocytosis of apoptotic prostate cancer cells via milk fat globule-EGF factor 8 (MFG-E8) promotes macrophage polarization into protumorigenic M2 phenotype [45]. Recently, another study confirmed that efferocytosis of apoptotic cancer cells by M2 bone marrow-derived macrophages displayed the pro-inflammatory phenotype when compared to peritoneal macrophages, suggesting that the bone is a fertile soil that supports skeletal metastasis [16]. Altogether, these studies have shed light on the crucial role of macrophage-mediated efferocytosis of apoptotic cancer cells in supporting tumor growth and metastasis in the bone. Further studies will determine if targeting the efferocytic machinery in M2 macrophages reduces the pro-inflammatory response upon apoptotic cancer cells clearance and inhibits tumor progression and metastasis in the bone.

### 2.3. Tumor-Associated Macrophages as Immunotargets and Their Potential Therapeutic Use in Bone Metastasis

Various therapeutic antibodies and molecules have been designed to target TAMs through different methods and they are used alone or in combination with other therapies. These therapies consist in depleting, inhibit recruitment and reprogramming of TAMs in the tumor microenvironment.

Two of the most studied therapeutic strategies to deplete TAMs from the tumor microenvironment are the inhibition of CSF-1/CSF-1R signaling and the use of liposomes containing clodronate. CSF-1R regulates macrophage differentiation, polarization, and migration of macrophages and hence CSF-1R signaling has been targeted by small molecules and antibodies to deplete macrophages in the tumor microenvironment. Pexidartinib (PLX3397), JNJ-40346527, and PLX7486 are tyrosine kinase inhibitors of CSF-1R signaling that are currently being used in phase I–III clinical trials for different types of cancers [155,156,157,158]. GW-2580, a CSF-1R inhibitor, has been used in acute myeloid leukemia patient samples. This study revealed that samples from de novo and low risk AML patients were more sensitive to GW-2580 treatment and samples from patients with reduced overall survival showed resistance to the CSF-R1 inhibitor [159]. Emactuzumab (RG7155) is a CSF-1R antibody that blocks CSF-1R dimerization in CSF-1R^+^CD163^+^ TAMs to deplete them from the tumor microenvironment, alone or in combination with other anti-cancer drugs [160,161]. Clodronate is a bisphosphonate that has been used as a therapeutic molecule since the 1960s due to its anti-inflammatory and analgesic actions. However, during the last years, liposomes containing clodronate (clodrosomes) have been studied as macrophage-depleting agents. Liposomes are taken by phagocytic cells, and once internalized clodronate molecules are released to promote macrophage apoptosis. Various studies have demonstrated that liposomes containing clodronate are taken up by macrophages which reduced macrophage tumor infiltration in bone and lung metastasis inhibiting metastasis progression in animal models [4,94,162]. Further clinical studies are needed to determine the doses and the use of these molecules/antibodies alone or combined with other anticancer therapies.

CCL2-CCR2 signaling is key during TAM recruitment to the tumor microenvironment; therefore, inhibition of CCL2 has been associated with reduced tumor progression in different cancer models, including bone [10,163]. Carlumab is a human antibody that binds to CCL2, which reduced tumor growth, infiltration of phagocytic macrophages and blood vessel density [164,165]. Unfortunately, no therapeutic efficacy was reported in a carlumab phase II clinical trial including castration-resistant metastatic prostate cancer patients [166]. PF-04136309 is a CCR2 inhibitor that, in combination with Folfirinox, resulted in local tumor control in pancreatic cancer patients [167]. Further studies are required to understand the effectiveness of the mechanisms that inhibit monocyte recruitment to the tumor microenvironment.

Macrophages are characterized by their plasticity; hence, therapeutic modulation of TAM polarization may be an efficient therapeutic strategy since this approach promotes their polarization towards the immunostimulatory M1 phenotype instead of depleting all macrophages from the tumor microenvironment. Toll-like receptors play a key role during innate immune responses and have dual roles in tumor promotion and inhibition in cancer [168,169,170]. Macrophage TLR3, TLR7, TRL8, and TLR9 drive antitumor immune responses. Poly I:C is a TLR3 agonist that stimulates M1 polarization in macrophages in vitro and in vivo [171,172,173,174]. Resiquimod (R848) is a potent TLR7 and TLR8 agonist that triggers antitumor responses [175,176]. A clinical study showed that TLR9 ligand (IMO-2055) has possible antitumor activity when combined with erlotinib and bevacizumab in advanced or metastatic non-small cell lung cancer patients [177]. Another method uses oligonucleotide (mRNA, siRNA, and miRNA) delivery techniques to reprogram TAMs such as charge-altering released transporters (CARTs) and other nanoparticles. Biodegradable nanoparticles are also being used to deliver mRNAs encoding the interferon regulatory factor 5 (IRF5) and IκB kinase β (IKKβ), to decrease the expression of M2-like genes such as Serpinb2 and CCL11 and increase the secretion of M1-like cytokines such as IL-12, IFN-γ, and TNF-α in ovarian cancer, glioma, and lung metastasis mouse models [178]. Nanoparticles carrying miRNA-155 have also been used to reprogram TAMs towards the M1 phenotype [179].

CD47 expressed by tumor cells binds to signal regulatory protein α (SIRPα) on TAMs suppressing tumor cell phagocytosis. Studies showed that CD47 blockade promotes macrophage reprogramming, which drives macrophage phagocytosis of cancer cells in xenograft mouse models [180,181]. Hu5F9-G4 and CC-90002 are CD47 antibodies that are currently being studied in phase I clinical trials with promising results [182]. TTI-621, a recombinant protein that blocks the CD47-SIRPα signaling in humans, is currently being studied in clinical trials including multiple solid tumors based on previous xenograft studies where it improved macrophage phagocytosis of cancer but not normal cells [183]. CD40-CD40L signaling polarizes M2 TAMs to M1 and increases IL-12 expression that promotes the maturation of T helper and cytotoxic cells. CP-870,893 is an agonistic anti-CD40 antibody utilized in clinical trials, where patients treated with CP-870,893 showed an improvement in antitumor activity in different types of cancers [184,185,186].

Despite the detrimental effect of skeletal metastases, as for other types of metastases, no therapeutic strategies are available to cure this disease [41]. However, few animal studies have used TAM depletion mechanisms to treat metastatic lesions. For instance, monocyte chemo-attractant protein 1 (CCL2) is involved in the recruitment of macrophages to the tumor microenvironment through activation of the CCR2 receptor [187]. In breast and prostate cancers elevated CCL2 serum levels correlate with advanced stages of the disease, suggesting a link between CCL2 expression and bone metastasis [188,189]. Cathepsins have an important role during tumor cell invasion and metastasis, and cathepsin K (CTSK) secreted by TAMs plays a key role in promoting prostate cancer skeletal metastasis [190]. Moreover, TAM and osteoclast depletion by clodronate liposomes (CI2MDP-LIP) showed a reduction in the number and size of bone metastatic lesions [162]. In addition, anti-CD115 antibody (CSF-1R antibody) treatment depletes TAMs decreasing bone lesions in a breast cancer mouse model [191]. Increased expression of hyaluronan synthase 2 (HAS2) in metastatic tumor cells is crucial for the interaction of tumor cells and macrophages in bone, this interaction increases PDGF secretion in macrophages, which promoted stromal cells activity and cancer cell self-renewal [192]. Interestingly, intratibial inoculation of murine prostate cancer cells (RM1, cell line) into macrophage Fas-induced apoptosis (MAFIA) mice (lacking M2 macrophages) resulted in decreased osteolysis in bone compared to vehicle-treated controls [4,42].

Macrophage-dependent efferocytosis of apoptotic cancer cells drives NF-κB and Stat3 transcriptional machinery to promote pro-inflammatory cytokine production that may lead to skeletal metastases [44]. Macrophage reprogramming immunotherapies have been suggested as an attractive approach to target the efferocytic machinery that supports metastatic prostate cancer [193]. Therefore, characterization of the molecular mechanisms involved in macrophage efferocytosis of cancer cells is a crucial step to identify novel drug targets to be used as coadjuvant therapies to treat skeletal metastases. In contrast, TAM depletion in bone metastasis is a complicated approach since these therapies may target also osteoclasts. Future studies need to use a better therapeutic approach that targets TAMs, MAMs, or their detrimental downstream signaling exclusively.

Here, we discuss the role of macrophages in supporting tumor growth and metastases, shedding light on TAMs/MAMs as promising targets for skeletal metastases immunotherapies.

## 3. Role of T Cells in Bone Metastasis

While macrophages and innate immunity are critical to protect us during the first hours and days of an infection, their response is not specific, and more time is needed to mount a specific response through adaptive immunity. This response is based on the recognition of antigens, usually small peptides, by lymphocytes through their antigen receptors: the B-cell receptor or BCR on B cells, and the T-cell receptor or TCR on T cells. Both cell types are critical during the development and progression of cancer, as well as during anti-cancer therapy. Here, we focus on the role of T cells in cancer, bone metastases, and immunotherapy.

### 3.1. T Cells

T cells originate from hematopoietic stem cells and lymphoid progenitors that are stored in the bone marrow. They continue their differentiation early on in the thymus and then are stored in secondary lymphoid organs such as the lymph nodes waiting to be activated by the presentation of antigens. Many different subsets of T cells exist to tackle the many different microbes we are exposed to, and a first classification of T cells is based on the composition of the heterodimeric TCR.

The large majority of human T cells are alpha beta (αβ) T cells with their TCR made of α and β chains. These are referred to here simply as T cells. These conventional T cells do not directly recognize the antigens. The TCRs only recognize antigens when they are loaded onto and presented to the TCRs by the major histocompatibility complex (MHC) molecules. MHC, also called H-2 antigens (histocompatibility-2 antigens) in mice and HLA antigens (human-leucocyte-associated antigens) in humans, can be separated into two classes. Class I MHC (MHC-I) are expressed by all cells in our body and bind to TCRs associated with the co-receptor CD8, on CD8^+^ T cells, while Class II MHC (MHC-II) are only expressed on antigen presenting cells (APC), such as dendritic cells (DC) or macrophages, and bind to TCRs associated with CD4, on CD4^+^ T cells [194]. After activation, and based on the cytokines they will be exposed to, naïve CD4^+^ T cells (Th0) can polarize into other subsets such as the classical T-helper 1 (Th1), 2 (Th2), or 17 (Th17), or induced T-regulatory cells (iTreg) [195]. CD4^+^ T cells are critical to the establishment of an effective and well-regulated immune response during an infection, as well as during cancer. CD8^+^ T cells are crucial during intracellular infections (i.e., viruses and intracellular bacteria) and cancer. Cytotoxic CD8^+^ T cells or lymphocytes (CTL) that release cytotoxic molecules (i.e., perforin and granzymes) and IFNγ are the most well-known and also called Tc1, as they are only one of the many subsets of CD8^+^ T cells that also include Tc2, Tc9, Tc17, and CD8^+^ T regulatory (Treg) [196,197].

Another group of T cells is the gamma delta (γδ) T cells or unconventional T cells whose TCR is composed of γ and δ chains. Unlike αβT cells that depend on the MHC to recognize antigens, the γδT cells are not MHC restricted, meaning that their TCR can directly recognize antigens, in absence of MHC, as an antibody does [198,199]. In addition, while αβ TCRs recognize peptides, γδ TCRs recognize non-peptidic antigens.

This diversity of T cell subsets allows adapting the immune response to the nature of the infection (i.e., intra- or extracellular bacteria, virus, and parasite). Another key determinant is the diversity of the TCR repertoire and the ability to generate a large amount of T cells that recognize different antigens [200]. This diversity is reached through a stochastic DNA recombination of the variable (V), diversity (D), and joining (J) segments of the TCR genes that is combined with the insertion and deletion of nucleotides, a process called VDJ recombination [201]. The randomness associated with these rearrangements generates a large variety of sequences in the different T-cell clones, and a multitude of TCRs that recognize foreign antigens. Due to this randomness, some of the TCRs generated will recognize antigens derived from the host proteins or self-antigens derived from the host proteins. To prevent the persistence of these T cells that would target host cells, and avoid autoimmune reaction, the central and peripheral tolerance ensures that these T-cell clones are eliminated or turned into Tregs that also prevent autoimmunity [202]. Similar mechanisms also ensure that BCR and antibodies recognizing self-antigens are not conserved. Since our adaptive immunity is trained to ignore our cells, it makes it more challenging, albeit not impossible, for T cells to recognize cancer cells derived from normal cells.

### 3.2. Anti-Cancer Response of T Cells

From the moment of fertilization, cells start accumulating mutations due to endogenous (i.e., errors in DNA replication) or exogenous factors (i.e., sunlight, radiation, and smoking). Since only ~1.5% of the genome contains protein-coding information, somatic mutations are likely to not have any effect, and are referred to as passenger mutations, but with time some driver mutations can occur and, although they represent less than 5% of the mutations, they can cause the transformation of normal cells to tumor cells [203,204]. Driver or passenger mutations can lead to the formation of neoantigens, specific to tumor cells that activate a specific immune response and T cell infiltration in the tumor [205]. Mutations are not the only source of tumor-specific antigens (TSA). Viruses were detected in ~17% of the datasets of the Pan-Cancer Analysis of Whole Genomes Consortium, mostly human papilloma (HPV), hepatitis B (HBV), and Epstein-Barr viruses (EBV) [206]. Viral proteins such as E6 and E7 for HPV or EBNA-1 for EBV can lead to the detection of viral antigens, and a specific immune response [207,208]. Other antigens, the tumor-associated antigens (TAA) are found in the tumor cells and in the normal cells, but due to aberrant expression in space, time, or quantity can make cancer detectable. For example, differentiation antigens such as the cancer testis antigens expressed in the immune privileged testis can be immunogenic when they are expressed in tumor cells, as in melanoma, even if they are not mutated [209].

**Figure 3:**
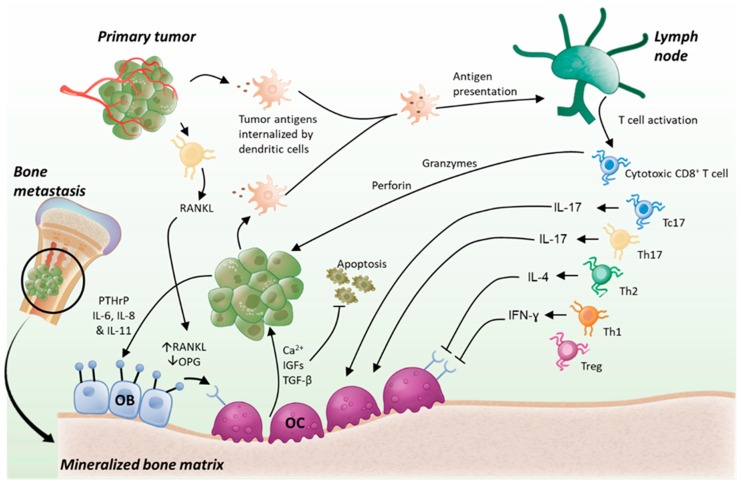
The role of T cells in bone metastasis. Antigens from dead cancer cells are taken up by dendritic cells to be presented to T cells in lymphoid organs such as the lymph nodes. Primed T cells can then home to the tumor site. While cytotoxic CD8^+^ T cells can induce the apoptosis of cancer cells, local factors, such as bone-derived TGF-β or bone marrow Tregs inhibit this cytotoxic response. TGF-β is released due to the bone resorption by osteoclasts (OC) caused by cancer cells cytokines (i.e., PTHrP, IL-6, IL-8, or IL-11), and the subsequent expression of RANKL by osteoblasts (OB). T cells that produce IL-17 (Th17 or Tc17) or RANKL (Th17) can also increase bone resorption and support the development of bone metastases, while IFN-γ and IL-4 derived from Th1 and Th2, respectively, can inhibit osteoclast formation and potentially limit bone metastases.

To obtain T cell activation and an anti-tumor response, a series of steps and interactions, referred to as the cancer-immunity cycle, is necessary [210]. Briefly, tumor antigens released from live or dead cancer cells are picked up by antigen presenting cells (APC) such as dendritic cells (DC), processed and presented on their MHC molecules. DCs then migrate to lymph nodes, looking for T cells with a TCR recognizing the antigens presented to prime and activate them (Figure 3). As such, the larger is the diversity in the TCR repertoire, the better is the protection against cancer cells [200]. Activated T cells start proliferating and traffic back to the tumor to infiltrate it. A strong infiltration of T cells within the tumors is a critical step, as it correlates with better patient survival in different types of cancers and in the case of breast cancer with a better response to neoadjuvant therapy [211,212,213,214]. After recognizing their cognate antigen, T cells, typically CD8^+^ T cells, kill cancer cells by releasing cytotoxic proteins such as perforin and granzymes from cytotoxic granules or by expressing Fas ligand that can engage the death receptor Fas on cancer cells. Such ability of T cells to kill cancer cells can now be assessed in real-time imaging, microfluidic systems that can help to identify characteristics and markers of the most efficient anti-cancer T cells [215,216]. In addition to cytotoxic T cells, CD4^+^ T cells also infiltrate the tumors and are key in the response to cancer, by coordinating the activation and function of the CTL against cancer cells [217,218,219].

Killing of cancer cells via T cell driven processes, if efficient, will lead to the elimination of the tumor. However, some cancer cells have the means to eventually survive and may then enter in a latency or equilibrium phase during, which the immune surveillance prevents the development of the tumor while not eliminating it. Koebel et al. [220] elegantly demonstrated how the proliferation of stable fibrosarcoma tumors in mice was resumed when T cells were depleted or transplanted in immunodeficient mice. Ultimately, cancer cells can escape from the control of the immune response and different mechanisms have been characterized [221]. Cancer cells can eventually lose the expression of the MHC-I, and in the absence of antigen presentation they then avoid the cytotoxic effect of T cells. Through genetic instability, some cancer cells may lose the expression of the antigenic protein and hence the negative selection of the immune system [222,223]. Besides turning invisible to the immune system, cancer cells can inhibit the immune response either by directly producing immunosuppressive factors, such as the immune checkpoint molecule PD-L1, or recruiting cells such as the cancer-associated fibroblasts, Tregs, M2 polarized macrophages or myeloid-derived suppressor cells (MDSC) that secrete immunosuppressive cytokines, such as IL-10, TGF-β, or VEGF, supporting then the growth of the tumor [210,221].

All these interactions between the cancer cells and the immune system compose the cancer immunoediting or immunoedition process, with its three steps or three Es: elimination, equilibrium. and escape. It is a major focus in cancer research and treatment [224], and its entrance among the hallmarks of cancer [225] highlights how it applies to most if not all forms cancers, including bone metastases.

### 3.3. Role of T Cells in Bone Metastasis, Friend or Foe

As in the primary tumor or other metastatic sites, the immune system and T cells should specifically target cancer cells in bone metastatic sites. This is especially likely when considering the importance of the bone marrow as a lymphoid organ in immunological memory, a hallmark of the innate immunity. Once T cells have been activated by an antigen, some of them will remain as memory T cells that can be re-activated in the case of a second encounter with these antigens. The bone marrow serves as a reservoir for memory CD4^+^ T cells [226,227] and memory CD8^+^ T cells [23,228], where they seem to be attracted by bone-derived cytokines, such as IL-7, and, interestingly, CXCL12, similar to cancer cells. Therefore, considering the time necessary for a tumor to grow and cancer cells to reach bone and form a bone metastasis, it is reasonable to hypothesize that some memory T cells recognizing tumor antigens are present in the bone marrow. Feuerer et al. [229] found that there is an increased amount of memory CD4^+^ and CD8^+^ T cells in the bone marrow of patients with breast cancer compared to healthy ones. In addition, patients who had cancer cells in their bone marrow, based on the detection of the expression of cytokeratine 19, also had more memory T cells when compared to breast cancer patients with cancer-free bone marrow. Most importantly, some of the memory CD8^+^ T cells recognized antigens derived from cancer cells, including an antigen from the HER2/neu protein. The transfer of T cells isolated from the bone marrow of breast cancer patients and re-stimulated ex vivo was able to reduce the growth of tumor fragments from the same patient, transplanted in NOD/SCID mice [230]. A pilot study tested whether such memory T cells could be functional in patients. T cells were collected from the bone marrow of advanced breast cancer patients and co-cultured with DCs pulsed with antigens from the breast cancer cells MCF-7 before transferring them to back to the patients [231]. One week after the transfer, it was possible to detect T cells producing IFN-γ in response to tumor antigen in the blood of half of the patients (6 of 12), referred to as a type-1 response, showing that these transferred T cells could persist for a while and target cancer cells. Interestingly, 85% of the patients without bone metastases had detectable circulating T cells with a type-1 response, while none of the patients with bone metastases had. It remains to be determined whether this lack of tumor antigen-reactive type-1 T cells was due to an effect of the bone metastases on the T cells before or after the transfer. That would indicate whether patients with bone metastases are eligible or not for such therapy.

In some of the patients with bone metastases, it was at least possible to detect a type-2 response: some T cells secreted IL-4 in response to cancer cell antigens [231]. Such type-1 and -2 responses are important as they could be beneficial for the treatment of bone metastases. IFN-γ produced by Th1 or CTLs can inhibit the development of cancer by increasing the expression of the MHC-I, hence making cancer cells more detectable by CTLs [232], or by directly inhibiting cancer cell proliferation or inducing apoptosis [233]. When it comes to cancer and bone, IFN-γ is an important regulator. In HTLV-1-Tax transgenic mice, the expression of the viral oncoprotein Tax, from human T-cell leukemia virus type 1, causes the spontaneous development of soft tumor and osteolytic bone metastases [234]. In the absence of IFN-γ, in *Tax^+^IFNγ*^−/−^ knockout mice, osteolytic lesions were increased. Although it was not clear what the involvement of the adaptive immune system was in this model, IFN-γ had a direct anti-cancer effect by inhibiting the proliferation and inducing the apoptosis of tumor cells [234]. In addition, the presence of IFN-γ decreased tumor associated bone loss and the formation of osteoclasts (Figure 3). Such an anti-osteoclastic effect of IFN-γ correlates with an independent study where Th1 cells prevented the formation of osteoclasts in vitro [235]. This effect was mediated by IFN-γ since the anti-osteoclastic effect of Th1 cells was prevented when using osteoclast precursors from mice deficient in the IFN-γ receptor (*Ifngr1*^−/−^). Th1 cells could then be beneficial for patients with bone metastases or myeloma due to their combined anti-osteoclastic and anti-cancer effects. In a 5TGM1 mouse myeloma model, Th1 cells recognizing an antigen from the specific immunoglobulin or idiotype secreted by myeloma cells caused the lysis of 5TGM1 cells in vitro through FasL–Fas interaction. Treatment with such idiotype-specific Th1 cells prevented tumor growth and the death of three out of five mice previously inoculated with myeloma cells [236].

Similarly, Th2 cells also inhibited the formation of osteoclasts in vitro but not when using bone marrow cells from *Stat6* knockout mice (*Stat6*^−/−^), a key transcription factor downstream of IL-4 and its receptor, confirming that IL-4 mediates the anti-osteoclastic effect of Th2 cells (Figure 3) [235]. Unlike Th1, the effect of Th2 in myeloma remains unclear. Idiotype-specific Th2 cells did not have any effect against 5TGM1 myeloma cells in C57BL/KaLwRij mice [236], however, when transferred in SCID mice, they prevented the growth of MOPC135 myeloma cells and mouse death. These Th2 cells provided long lasting immunity as seen by cancer cells being eradicated in mice when inoculated more than 40 days after the initial treatment [237].

Similar work against bone metastases from breast or prostate cancer does not seem to have been attempted thus far, possibly due to the difficulty in identifying specific antigen and anti-cancer T cells or the limitation imposed by the available pre-clinical models. Most models of bone metastases from solid tumors are based on the inoculation of human cancers cells into immunodeficient mice, such as nude or SCID mice that lack T cells [238,239]. Therefore, to assess the role of the adaptive immunology in bone metastases, scientists have had to rely mostly on the inoculation of cancer cells derived from inbred mice into mice of the same breed. One of the cell lines often used for immunological study is the B16 melanoma cell line, derived from C57BL/6 mice and its different subclones (i.e., B16-F1 and B16-F10) that can cause bone metastases [240,241]. With such model, Zhang et al. [242] demonstrated that the development of B16 bone metastases was increased in athymic nude mice lacking T cells, suggesting that T cells were limiting the development of bone metastases in normal C57BL/6 mice. Similarly, in mice with a knockout of MHC-I or MHC-II that lack CD8^+^ and CD4^+^ T cells, respectively, there was an increase of the skeletal tumor burden compared to wild-type mice, suggesting that both CD4^+^ and CD8^+^ T cells have a protective effect against bone metastasis [242].

Despite such encouraging evidence in myeloma and melanoma models, other data suggest that T cells may not be able to target cancer cells or may eventually increase bone metastases. One syngeneic model for bone metastases uses 4T1 breast cancer cells, derived from Balb/C mice, and that can spontaneously form bone metastases from an orthotopic tumor (although the timeline of the formation requires surgical removal of the tumor to have enough time for the bone metastases to develop) or after intra-cardiac inoculation when inoculated in Balb/C mice [239,243]. Using this model, Monteiro et al. [244] characterized how a 4T1 tumor in the mammary fat pad of mice supported a pre-metastatic niche where expansion of a CD4^+^ T cell population caused an increase of bone resorption before the 4T1 cancer colonized the bone marrow. Interestingly, these CD4^+^ T cells produce IL-17 that increases osteoclastogenesis in vitro [235]. In vivo, subperiosteal inoculation of lipopolysaccharide (LPS) causes T cell-dependent inflammation and the recruitment of osteoclasts, which was prevented in IL-17 deficient mice (*Il-17*^−/−^). Th17 cells are also critical in the development of autoimmune arthritis, as IL-17 increases the osteoblastic expression of RANKL and subsequently the differentiation of osteoclasts with synovitis and joint destruction [245,246,247]. However, silencing of IL-17 in 4T1-primed T cells did not reverse the induction of osteoclastogenesis. The T cell-dependent induction of osteoclast formation was reversed only when RANKL was reduced in T cells, preventing the T cell-mediated increase of metastatic colonization in mice (Figure 3) [244]. In cancer patients, circulating T cells were found to increase the formation of osteoclasts. T cells in the blood of patients with multiple myeloma induced osteoclastogenesis ex vivo by producing RANKL [248]. Similarly, T cells from peripheral blood mononuclear cells (PBMCs) of patients with solid tumors and osteolytic bone metastases increased spontaneous osteoclastogenesis, in the absence of M-CSF or RANKL in the culture media, which was not possible with PBMCs of healthy donors [249]. Unlike with T cells from myeloma patients, this osteoclastogenesis was not reversed when neutralizing RANKL with OPG, but only when adding a TNF-α-neutralizing antibody [248].

Overall, these results show that the role of T cells on osteoclasts and the development of bone metastases or myeloma can vary, from T cells being suppressors to promoters of the pathology. It remains unclear what the factors are that trigger one behavior or the other. Similarly, in patients, the effect of the immune system and of immunotherapy varies greatly, leading to the discrimination between “hot” and “cold” tumors and the Immunoscore to classify them [250,251]. While hot tumors are likely to elicit a spontaneous or immunotherapeutically-induced immune response against cancer cells, cold tumors are characterized by a low or absent immune response. Such a phenotype seems as much defined by the cancer cells themselves as by the tumor microenvironment and its effects on the immune response.

### 3.4. Effect of the Bone Metastatic Microenvironment on T Cells, Action-Reaction

It remains to be directly determined whether cancer cells in bone are hot or cold tumors but there is an increasing amount of indirect evidence that suggests that the bone marrow is likely to be cold for the anti-cancer immune response.

#### 3.4.1. Bone Marrow Mesenchymal Niches and Resident Memory T Cells

Among its many functions, bone and the bone marrow induce the differentiation of CD4^+^ and CD8^+^ T cells from effector to memory T cells, under the control of IL-7 [252,253]. Circulating levels of IL-7 are increased in patients with solid tumors compared to healthy patients; they are also higher in cancer patients with bone metastases than patients without bone metastases [254,255,256]. Such mechanisms could explain why the amount of memory T cells is increased in the bone marrow of breast cancer patients with and without bone metastases [229]. In addition, stromal cells in their niches in close proximity to memory T cells suppress the proliferation of memory T cells, and/or induce their apoptosis to prevent their activation [257,258,259]. It is therefore possible that the bone marrow of patients with bone metastases or myeloma turns T cells into memory T cells, and maintains them in such state, preventing an effector function against cancer cells.

#### 3.4.2. Cell- and Bone-Derived Transforming Growth Factor-β (TGF-β)

TGF-β is one of the most potent immunosuppressors. As reviewed by Batlle and Massagué [260], TGF-β regulates all the major steps of the immune response: from the function of dendritic cells and the presentation of antigens to the induction of Treg differentiation, as well as the function of Th1, cytotoxic T cells and natural killer cells. These effects are important for the regulation of a normal immune response, as well as during pathologies such as autoimmune diseases or in cancer development. In the tumor microenvironment, different cell types, such as Tregs, produce TGF-β; in bone metastases, TGF-β can also be released from the bone matrix in response to osteolytic tumor incitement.

Tregs or regulatory T cells are key players that prevent autoimmune reactions by inhibiting T cells detecting self-antigen that were not eliminated during the tolerance process. Production of TGF-β by Tregs is key in preventing autoimmune reactions. As such, the selective knockout of the type 2 TGF-β receptor in T cells (*Tgfbr2^fl/fl^*xCD4-Cre) led to mouse death by three weeks of age as Tregs failed to inhibit autoimmune reactions [261]. In cancer, infiltration of Tregs in the tumor leads to a decreased anti-cancer response and reduces the survival of patients with different types of cancer, including breast cancer [261,262]. The bone marrow naturally contains a large amount of Tregs [263], which could further increase during cancer. In prostate cancer patients, the number of functional Tregs in the bone marrow is increased when bone metastases are present [264]. What remains puzzling is that Tregs seem to suppress osteoclast formation and bone resorption [264,265]. Transgenic mice overexpressing the transcription factor FoxP3, critical for the development of Tregs, have increased numbers of Tregs and increased bone mass due to reduced osteoclasts and bone resorption, while bone formation was unchanged [265]. Despite these preclinical findings, patients with bone metastases, whether from breast cancer that tend to be osteolytic or from prostate cancer usually osteoblastic, have elevated bone resorption as indicated by bone turnover markers [266,267,268]. Hence, it is unclear whether Tregs dispose of all of their functions in bone metastases.

Another major source of TGF-β in bone is from the mineralized bone matrix. Using a bioluminescent model where the expression of luciferase is under the control of the TGF-β signaling pathway, Korpal et al. [269] found that TGF-β signaling is activated in breast cancer cells in murine bone metastases, and that inhibition of bone resorption with a bisphosphonate decreased the corresponding luciferase signal, confirming that TGF-β released from the bone during osteoclastic resorption is the main source of TGF-β for cancer cells in bone. This model used nude mice that lack T cells, and it remains to be demonstrated whether bone-released TGF-β really inhibits the immune response in bone metastases, and whether inhibitors of bone resorption like bisphosphonates or RANKL-neutralizing antibodies could fully or partly reverse this effect. Other methods to inhibit TGF-β signaling such as small molecule inhibitors of the TGF-β type 1 receptor (TGFBR1), TGF-β neutralizing antibody, and oncolytic viruses producing a soluble form of TGFBR2 (sTGF-βRII-Fc) have also been found to be efficient at inhibiting bone metastases [270,271,272,273], and could also be efficient at inhibiting TGF-β-mediated immunosuppression. Using the 4T1 syngeneic breast cancer model, intratumoral delivery of an oncolytic adenovirus expressing sTGF-β-Fc reduced the amount of Tregs in the tumor, while increasing the infiltration of CD8^+^ T cells and increasing the expression of T cell cytotoxic factors, and caused a reduction of tumor growth [274]. Similarly, combination of the antineoplastic drug cyclophosphamide with the pan-TGF-β neutralizing antibody 1D11 reduced the growth of 4T1 tumors and lung metastases in mice while increasing tumor-infiltration of T cells producing IFN-γ [275]. Although 1D11 also inhibited bone metastases from 4T1 cells in Balb/C mice [276], it was not assessed whether there was an effect on T cell infiltration within the bone or a change in T cell-mediated cytotoxicity.

#### 3.4.3. Myeloid-Derived Suppressor Cells

Overall, cancer cells dispose of a large range of possibilities to inhibit the immune response, whether by producing their own immunosuppressive factors or by recruiting immunosuppressive cells, such as Tregs, cancer-associated fibroblasts (CAFs), M2 macrophages, and myeloid-derived suppressor cells (MDSC).

Regular myeloid cells, such as monocytes, macrophages, granulocytes, or dendritic cells are key for our immune protection, and their differentiation is well regulated by cytokines such as the G-CSF and the M-CSF for granulocytes and macrophages, respectively. However, it seems that the overproduction of these factors during pathologies such as cancer result in immature forms of myeloid cells with potent immunosuppressive functions accumulating in mice and in patients [277]. Increased levels of MDSCs were detected in the blood of patients with breast [278,279] and prostate cancer [280,281], and correlated with the tumor stage. MDSCs can also be used a prognostic marker as evidenced by breast and prostate cancer patients having more elevated levels of circulating MDSCs and decreased overall survival [279,280,281]. Thus far, two different subpopulations of MDSCs have been characterized: the monocytic MDSC (M-MDSC) and the polymorphonuclear MDSCs (PMN-MDSC) that appear to derive from the monocytic and the neutrophil branches of myeloid cells, respectively [277].

The mechanisms of MDSC-mediated immunosuppression are varied and differ between sub-populations. M-MDSCs have elevated levels of inducible nitric oxide synthase (iNOS), while PMN-MDSCs express arginase 1 (ARG1), leading to the production of nitric oxide (NO) and reactive oxygen species (ROS), respectively, and the subsequent inhibition of T cells [282]. MDSCs can also induce the differentiation or recruitment of Tregs and produce the same immunosuppressive cytokines as Tregs, such as TGF-β and IL-10. Another interesting mechanism is through the sequestration of the amino acids arginine (L-Arg) and cysteine that T cells cannot produce. MDSCs expressing the cationic amino acid transporter 2B (CAT-2B) import L-Arg in their cytoplasm where is it used as a substrate by ARG1, resulting in depletion of arginine in the tumor microenvironment and T cell neutralization [283]. Similarly, T cells lack cystathionase to convert methionine into cysteine and the x_c_^-^ transporter to import cystine, disulfide-bonded cysteine. They rely on DCs to directly supply the cysteine necessary for their activation and function [284]. MDSCs express the transporter x_c_^-^, and internalize cysteine but do not secrete cysteine afterward, leading to its depletion in vitro. Subsequently, 4T1 tumor-bearing mice have reduced serum levels of cysteine. T cell inhibition mediated by MDSCs is then reversed by the addition of cysteine in the media [284].

In breast cancer models of bone metastases, as well as myeloma, there is an increase of MDSCs in the bone marrow of mice [285,286,287]. Transfer of MDSCs from tumor-bearing mice increases the development of bone metastases. However, this increase is independent of T cell suppression, since it occurs in immunodeficient mice inoculated with human breast cancer MDA-MB-231 cells [285]. MDSCs co-cultured with MDA-MB-231 induced an increase of the expression of pro-osteoclastic factors such as PTHrP and Gli2 [286]. In addition, MDSCs from the tumor microenvironment can further differentiate into functional osteoclasts, explaining why the pro-bone metastatic effect can be independent of T cells in these models [285,286,287]. Zhang et al. [242] observed that B16 melanoma bone metastases were increased in mice deficient for the gene PLCγ2 (*Plcγ2*^−/−^) expressed in myeloid cells, despite an absence of osteoclasts. This effect could be explained by an interference with the function of MDSCs that also express PLCγ2, and were less potent at inhibiting CD8^+^ T cells [242]. Such results suggest that MDSCs could inhibit T cells in the bone metastasis microenvironment as well.

Different approaches have been tested to target MDSCs including inhibitors of the phosphodiesterase-5 (PDE5) (i.e., sildenafil, and tadalafil) [288,289], or the activation of the liver-x nuclear receptor/ApolipoproteinE signaling pathway with the agonist RGX-104 [290] that can decrease the levels of MDSCs in cancer patients. The chemokine receptor CXCR4 involved in the trafficking of hematopoietic stem cells and cancer cells to bone is also expressed on MDSCs, and the CXCR4 antagonist AMD3465 decreased the intra-tumoral infiltration of MDSCs, increasing the survival of mice that received an intratibial inoculation of 4T1 breast cancer cells [291,292]. The bisphosphonate zoledronic acid in combination with a DNA vaccine also decreases the number of circulating MDSCs and the volume of mammary carcinoma in FVB×BALB-neuT mice [293].

Thus, MDSCs are relevant therapeutic targets for the treatment of bone metastases, especially considering that the immunosuppressive properties of MDSCs are increased by hypoxia [294], and that bone and bone metastases are hypoxic microenvironments [295].

#### 3.4.4. Hypoxia-Mediated Immunosuppression

Low physiological oxygen pressures (pO_2_) cause a state of hypoxia that has been found in almost all types of cancer, making it one of the hallmarks from Hanahan and Weinberg [225]. Hypoxia is also important in bone where the pO_2_ can be as low as 1% in the bone marrow or for osteocytes in the bone matrix [296]. As a consequence, hypoxia and the induced-expression of the transcription factor hypoxia inducible factor-1α (HIF-1α) support the development of bone metastases, especially when combined with TGF-β signaling [295]. Hypoxia in the tumor microenvironment also regulates the immune response [297].

Due to their energetic needs and the hypoxia in the tumors, cancer cells need to adapt their metabolism, including glucose metabolism as they favor glycolysis, a phenomenon also known as the Warburg effect [298]. To fulfill their needs in glucose, cancer cells have increased expression of the glucose transporters GLUT1 and GLUT3, which can lead to a competition between the cancer cells and T cells for glucose uptake [299]. As a consequence of this glucose deprivation, the function of T cells is affected, leading to their anergy or to their apoptosis, and loss of effector function, such as decrease of granzyme B, perforin, and IFN-γ for cytotoxic CD8^+^ T cells [300]. Another consequence of this glycolysis is the production and secretion of lactic acid that can decrease the pH to 5.8–6.5 in hypoxic zones and inhibit T cell function [301]. Hypoxia-induced lactate and acidosis also decreases the proliferation and activity of T cells in the tumor microenvironment [302,303,304]. Among the different mechanisms identified, hypoxia induces the shedding and solubilization of the MHC class I chain-related molecule A (MIC-A) [305]. MIC-A is critical for the activation of NK cells and the elimination of cells that do not express the MHC-I. When present in a soluble form, MIC-A can also prevent effector function of antigen-activated T cells [306]. Such mechanisms can protect cancer cells from the immune response in tumors, and could also apply to bone metastases.

### 3.5. T Cell-Directed Immunotherapies and Their Possible Use for the Treatment of Bone Metastases

Different molecular and cellular mechanisms drive tumor growth, making it particularly challenging to find the most suitable treatment for each patient despite that their adaptive immunity has the ability to develop an anti-cancer immune response, specific for their cancer cells. However, as mentioned above, cancer immunoediting ultimately allows cancer cells to escape from the anti-tumor immune response, preventing the elimination of the tumor.

Understanding the interactions between cancer cells and the immune system allowed the development of multiple strategies of immunotherapies, some of which are having long-term beneficial effects in cancer patients. Unfortunately, not all of them work and that is the reason a new challenge is the identification of the most adapted immunotherapy for each patient and eventually for the treatment of bone metastases and myeloma. Blank et al. [307] considered seven parameters to obtain the largest amount of activated anti-cancer T cells within the tumor: (1) tumor foreignness; (2) general immune status; (3) immune cell infiltration; (4) absence of checkpoint; (5) absence of soluble inhibitors; (6) absence of inhibitory tumor metabolism; and (7) tumor sensitivity to immune effectors. Overall, if a tumor is presenting a small T cell infiltration, attention should be focused on mobilizing anti-cancer T cells to the tumor, while, in the case of larger but ineffective T cell infiltration, the treatment should focus on activating the immune response.

#### 3.5.1. γδT Cells and Nitrogen-Containing BPs

Although a lot of attention is oriented toward the conventional αβT cells, γδT cells could be just as relevant for the treatment of bone metastases or myeloma, especially for patients receiving nitrogen-containing bisphosphonates. As more potent bisphosphonates containing nitrogen atoms started being used in clinic, some patients started experiencing an acute-phase response, or flu-like symptoms and increased circulating levels of IL-6 or TNF-α [308]. Ex vivo, it was confirmed that γδT cells from the PBMCs were responsible for the secretion of these cytokines [309]. Nitrogen-containing bisphosphonates inhibit the farnesyl diphosphate synthase enzyme (FPPS) in the mevalonate pathway, causing the accumulation of its substrates, including isopentenyl diphosphate (IPP) that is recognized by the Vγ9Vδ2 T cells (also called Vγ9Vδ2 T cells) with the variable (V) gene combination Vγ9/Vδ2 as components of their TCR [310,311]. Cancer cells accumulating IPP can then be the target of the Vγ9Vδ2 T cells and their cytotoxic activity [312]. Since Vγ9Vδ2 T cells are restricted to humans and non-human primates, they are lacking in mice [313]. However, if Vγ9Vδ2 T cells are expanded from human PBMCs and transferred into NOD/SCID mice engrafted with breast cancer cells, they can decrease the volume of subcutaneous tumors [312] or of bone metastases [314] when mice were treated with a bisphosphonate. In patients, γδT also accumulates in the tumors and increased infiltration is associated with increased survival [315]. Hence, immunotherapy with transfer of γδT cells in cancer patients treated with bisphosphonate, in combination or not with IL-2, has been tested. Despite positive effects in a few patients, there were some significant side effects [316,317]. Therefore, current studies are attempting to further increase the anti-cancer effect of γδT cells using analogs of bisphosphonates that could be more efficient in patients [318,319].

#### 3.5.2. Immunotherapy Using Immune Checkpoint Inhibitors

The immune response is tightly regulated to prevent a too-strong response that would be detrimental or that would last too long. As such, T cells can express receptors such as the cytotoxic T-lymphocyte-associated protein 4 (CTLA-4) or the programmed cell death protein-1 (PD-1). These receptors serve as checkpoints for the immune response and deactivate T cells when they interact with their ligands: CD80 and CD86 for CTLA-4, or PD-L1 and PD-L2 for PD-1. T cells that recognize tumor cell antigen often express these receptors as a consequence of the prolonged exposition to these antigens [320,321]. Monoclonal antibodies against CTLA-4, PD-1, or PD-L1 have been tested in clinical trials and found effective for some patients with advanced melanoma [322] or non-small cell lung cancer [323]. Unfortunately, while some patients obtain long-term benefit from these treatments, some others do not gain any benefit, and some studies have reported negative effects [324]. Biomarkers allowing identifying patients who would benefit from treatments with immune checkpoint inhibitors are therefore needed.

Levels of expression of PD-L1 in the tumors condition the effects of treatment against the PD-L1/PD-1 axis but also affect the efficacy of a combined treatment anti-PD-1 and anti-CTLA-4 [325]. Retrospective analysis of the efficacy of anti-CTLA-4, anti-PD-1, and/or anti-PD-L1 in more than 1600 patients, with 10 different types of cancer, revealed that immune checkpoint inhibitors were more efficient as the tumor mutational burden (TMB) or foreignness of the cancer was increased [326]. In patients with non-small cell lung cancer, the combined treatment anti-CTLA-4/anti-PD-1 was more efficient in patients with a higher TMB (≥10 mutations per megabase) compared to patients with a lower TMB [323]. A treatment with immune checkpoint inhibitors was also more efficient than chemotherapy in patients with a higher TMB [323].

Immune checkpoint inhibitors could be efficient for the treatment of bone metastases. In a mouse bone metastasis model, treatment with an anti-CTLA-4 antibody decreased the skeletal tumor burden in mice inoculated with B16 melanoma cells [242]. Melanomas have the highest TMB and are usually quite immunogenic [327], which could explain the efficacy of anti-CTLA-4 in this model. Considering that cancers that spread to bones such as breast and prostate cancer or myeloma or develop in bone such as osteosarcoma have a relatively lower amount of mutations in the cancer spectrum [327], such cancer patients may not be the best candidates for treatments with immune checkpoint inhibitors. Combination of an anti-PD-L1 antibody with a nanoparticle albumin-bound paclitaxel for the treatment of patients with advanced triple negative breast cancer patients only improved the progression-free and overall survival when patients were PD-L1 positive. Therefore, patients with bone cancer or bone metastases having lower TMB may not benefit from a treatment with immune checkpoint inhibitors. An exception may be lung cancer that is the third most prone cancer to form bone metastases after breast and prostate cancer [328]. A retrospective study of 58 lung cancer patients with bone metastases presented at the annual meeting of the ASCO suggest that treatment with a combination of immune checkpoint inhibitors and other therapies including bisphosphonate or Denosumab (anti-RANKL) is associated with a better survival [329]. However, further validation is required.

The efficacy of immune checkpoint inhibitor could also be increased through combined therapy. The effect of the neutralization of the PD-L1/PD-1 axis in pre-clinical cancer models is increased when combined with the MDSC inhibitor RGX-104 [290], the 1D11 anti-TGF-β antibody [330], or Galunisertib, an inhibitor of the TGBFR1 [331]. Considering the importance of TGF-β in the development of bone metastases [269], and how different inhibitors of the TGF-β signaling pathway inhibit bone metastases in mice [270,273], such targeting both the immune checkpoints and TGF-β could be more efficient for the treatment of bone metastases.

#### 3.5.3. Immunotherapy with Bispecific and Trispecific Antibodies

Standard antibodies recognize only one specific antigen and combination therapies would require giving two antibodies to the patient, further increasing the cost of such treatments [332]. Biotechnological advances, however, have permitted the development of new bispecific and trispecific antibodies that can recognize up to two or three different antigens, respectively [333]. Such molecules could render treatments more affordable. Ravi et al. [334] developed a bifunctional molecule composed of the ectodomain of the TGFBR2, to trap TGF-β, and of an antibody targeting CTLA-4 or PD-L1. These Y-traps were more efficient at decreasing the development of tumors from human melanoma or breast cells in humanized NSG mice than a single treatment or a combined treatment with conventional antibodies [334].

Many bispecific antibodies bind to CD3 of the TCR and to an antigen on the cancer cells, e.g., EpCAM on carcinomas such as breast and prostate cancer (Catumaxomab and Solitomab), Her/neu on breast cancer cells (Ertumaxomab), PSMA on prostate cancer cells (Pasotuxizumab), or BCMA (B cell maturation antigen) on myeloma cells (AMG420) [333]. They allow recruiting T cells and keeping them in close proximity of the cancer cells to exert their cytotoxic effect. These bispecific antibodies can be referred to bispecific T cell engagers (BITE). In the case of the presence of a Fc fragment, such as for Catumaxomab, the antibody can also recruit cells with Fc receptors, such as macrophages, dendritic cells, or natural killer cells, to help eliminate cancer cells or co-stimulate T cells [333]. In a phase I clinical trial, patients with metastatic castration-resistant prostate cancer were treated with Pasotuxizumab. Besides being well tolerated, one of the patients had a marked regression of bone metastases, as measured by PSMA PET/CT [335].

Wu et al. [336] developed a trispecific antibody that binds to CD3, CD28, and CD38. CD38 is expressed on lymphoid cells, such as B and T cells, and cancer cells deriving from this lineage, such as myeloma cells, and it can be used as a therapeutic target. CD3 and CD28 are expressed on T cells and antibodies binding to them have been used as agonist to activate them. The trispecific antibody efficiently mediated the lysis of different myeloma cell lines by T cells. The antibody was even more effective at driving T cell cytotoxicity when myeloma cells expressed CD28 [336], and it could potentially be used for the treatment of myeloma patients.

#### 3.5.4. Engineering of T Cells for Cancer Therapy

Immune checkpoint inhibitors or bispecific T cell engagers rely on the presence of endogenous, anti-cancer T cells that have infiltrated the tumor of the patients. This implies that antigen-presenting cells were able to uptake tumor specific or associated antigens and find T cells with the TCR recognizing them, and that after priming T cells make it to the tumor. Should this sequence of event not happen and lymphocyte infiltration be limited, a possibility is to transfer to the patients T cells.

Adoptive T cell transfer that was developed by Dr. Steven Rosenberg at the National Cancer Institute (Bethesda, MA, USA) is based on the isolation and selection of anti-cancer T cells from a fragment of tumor [337]. After expansion in the laboratory, T cells are re-transferred to the patient. This approach has allowed obtaining regression or a durable complete response in patients with melanoma [338], and, although it is less efficient in carcinoma, long lasting effects were also achieved in a breast cancer patient [339]. Unfortunately, this treatment has only been used on a limited number of patients due to the challenges in isolating and selecting T cells.

An alternative approach is to generate anti-cancer T cells from the “normal” T cells of the patients, as is done with CAR-T cells. T cells are collected from the blood of patients and transduced with lentiviral particles to express a chimeric antigen receptor (CAR). CARs are transmembrane proteins whose extracellular domain is made of a ligand-binding domain of a single-chain antibody (scFv) specific for a tumor antigen, and the cytosolic extremity is composed of fragments of activation and co-stimulatory domains of CD3ζ, and CD8, CD28, or CD137. Patients then receive these CAR-modified autologous T cells. The first CAR-T cells developed were targeting CD19, a pan-B cell marker still expressed in multiple forms of hematological cancers. The efficacy of CD19-directed CAR-T cells in early trial was such that they received the breakthrough designation from the FDA in 2016, and by 2017 they were approved for the treatment of acute lymphoblastic leukemia. Although myeloma is a hematological malignancy, myeloma cells do not express CD19, as they originate from plasma cells that lose CD19 when differentiating from B cells. Despite that, treatment of patients with refractory multiple myeloma using CD19-targeting CAR-T cells was attempted [340]. Two of the ten patients treated had an increased progression-free survival, and analysis of bone marrow biopsies showed an absence of myeloma cells, although, ultimately, one of them had a relapse in the form of a more aggressive and treatment-refractory myeloma. Despite that, this study shows that CD19-CAR-T cell therapy could be adapted for patients with myeloma. Alternative treatments are developed and tested by CARs recognizing other surface antigen on myeloma cells such as CD138, SLAMF7, the κ light chain, BCMA, or CD229 [341,342,343]. CAR-T cells could also be used for the treatment of carcinoma if the appropriate antigen is targeted. For prostate cancer, CARs binding to the prostate-specific membrane antigen (PSMA), the prostate stem cell antigen (PSCA), and EpCAM have been tested in preclinical and phase I clinical trials [344]. Antigens such as ErbB2, MUC1, cMet, or Mesothelin have been tested in mice and in phase I or II trials for the treatment of breast cancer [345].

CAR-T cells are definitely potent, thanks to the multiple activating and co-stimulatory domains within the cytoplasmic extremity. Their potency can actually be an issue, as CAR-T cells are associated with serious side effects, such as cytokine storms. Thus, methods are sought to control their activity [346]. New generations of engineered T cells are also being developed using gene-edition. The first clinical trials were recently performed with autologous transfer of T cells modified to express a synthetic TCR transgene that recognizes the cancer-testis antigen NY-ESO-1, while the genes for the α and β chains of the TCR (TRAC and TRBC) were deleted using CRIPS-Cas9 to prevent competition with the transgenic TCR, as well as the gene for PD-1 to prevent T cells deactivation [347]. The treatment was well tolerated in the three patients of this phase I clinical trial, and the engineered cells were still detected nine months after the transfer. Interestingly, two of the patients had refractory advanced myeloma, and the treatment caused a partial decrease of the number of target cancer cells.

These results highlight the feasibility and therapeutic potential of engineered cancer-specific T cells for the treatment of cancer, and their ability to eliminate cancer in the bone microenvironment. It remains to be determined in future clinical trials whether they can reduce or eliminate bone metastases and other forms of cancer in bone.

## 4. Conclusions

Currently, patients with skeletal metastases receive palliative treatments to alleviate their pain or improve patients’ quality of life, and there are no effective treatments that can cure the disease. Therefore, numerous studies focus on investigating the role of the immune cell mechanisms in the bone in order to have a better understanding of their impact in metastatic cell growth in the bone. In this review, we discuss the role of macrophages and T cells in bone metastasis and how immunotherapeutic strategies that target these cells in primary and metastatic tumors may be used in the treatment of skeletal metastases.

TAM reprograming is one of the most promising strategies to treat bone metastases. Simply depleting macrophages or their recruitment may have a negative effect on normal bone remodeling processes causing osteopenia in cancer patients. Interestingly, macrophage-dependent efferocytosis of apoptotic cancer cells emerges as a key process during the development of bone metastases; therefore, the identification of the molecular mechanisms involved in this process are crucial for the development of novel immunotherapies that target efferocytic macrophages in skeletal metastases. Similarly, immunotherapy aiming to activate anti-cancer T cells has received an increasing amount of attention and shows great promise for the treatment of different kinds of cancers. However, its efficiency depends heavily on the nature of the cancer cells and the tumor microenvironment so that, while some cancer patients benefit from immunotherapy, others do not respond. Despite promising data from pre-clinical models, it remains to be determined whether T cells will benefit patients with bone metastases.

As highlighted in this review, a comprehensive knowledge of the effect and function of macrophages and T cells in the bone microenvironment is crucial to develop effective therapies against bone metastases and give new hope to patients with skeletal metastasis.
